# Editorial Note

**Published:** 1940

**Authors:** 


### Editorial Note

**The Middlesex Hospital.**

For the first four months of the War the pre-Clinical Medical Students of the Middlesex Hospital worked in the University of Bristol. They and their teachers adapted themselves splendidly to the new environment and made themselves thoroughly popular guests. We are pleased to publish a letter which Professor Kirk has addressed to this *Journal.* These friendships, which the fortunes of war have opened up, go a long way to minimize the unpleasantnesses :—

" I wonder if you can find a little space in your *Journal* for this short message from the members of the Middlesex Hospital Medical School whose good fortune it was to sojourn in Bristol from September to December, 1939 ? A good many months have sped since, in obedience to the call of higher authority, we returned to our home at Mortimer Street, London, but the passing of time and the crowded experiences of subsequent events have not diminished one jot the happiness we have in recalling those days we spent as the guests of the good folk of Bristol. In particular we recall most gratefully the kind and gracious hospitality so many of us received in the homes of the members of the medical profession in your Western city.

" Those of us who had the responsibility of finding at such short notice billets for a family of 200 teaching staff and students felt a little staggered when we contemplated the difficulties which loomed ahead as our first reconnaissance party boarded the train at Paddington. Our fears were foolish and ill-founded. We had never been to Bristol, and we did not know its warm-hearted people. The understanding help and unfailing courtesy of everyone to whom we appealed for assistance in our time of need speedily dissolved our problems and difficulties.

" The ready response of many new-found friends who, undaunted by the inconveniences and readjustments inevitably involved, opened their homes to accommodate so many of our staff and students is something we shall never forget. When the honeymoon stage was over and our foibles and idiosyncrasies became all too apparent, the patient tolerance of our hosts and hostesses won our admiration.

" The readiness, too, with which the University Authorities and Teaching Staff accommodated us and made everything comfortable and pleasant for us in an academic sense, is also gratefully acknowledged. We think it can be claimed that the students of the Middlesex School not only lost nothing by their evacuation, but profited by some of the experiences through which they passed during their stay in Bristol. For example, many of them still talk of the ' Galenicals ' and the stimulus they received even in these pre-clinical days from several excellent meetings of that Medical Society. Our only regret is that our stay in your city came so abruptly to an end just when we were beginning to take root in our new environment.

" To all those friends who smoothed out our difficulties and so unselfishly supplied our needs we send our cordial greetings and grateful remembrance."

